# Development of a High-Throughput Method to Study the Inhibitory Effect of Phytochemicals on Trimethylamine Formation

**DOI:** 10.3390/nu13051466

**Published:** 2021-04-26

**Authors:** Lisard Iglesias-Carres, Lauren A. Essenmacher, Kathryn C. Racine, Andrew P. Neilson

**Affiliations:** 1 Plants for Human Health Institute, Department of Food, Bioprocessing and Nutrition Sciences, North Carolina State University, Kannapolis, NC 28081, USA; liglesi@ncsu.edu (L.I.-C.); kcracine@ncsu.edu (K.C.R.); 2 Department of Food Science and Technology, Virginia Polytechnic and State University, Blacksburg, VA 24061, USA; elauren7@vt.edu

**Keywords:** atherosclerosis, gallic acid, chlorogenic acid, microbiota, trimethylamine

## Abstract

Choline is metabolized by the gut microbiota into trimethylamine (TMA), the precursor of pro-atherosclerotic molecule trimethylamine N-oxide (TMAO). A reduction in TMA formation has shown cardioprotective effects, and some phytochemicals may reduce TMA formation. This study aimed to develop an optimized, high-throughput anaerobic fermentation methodology to study the inhibition of choline microbial metabolism into TMA by phenolic compounds with healthy human fecal starter. Optimal fermentation conditions were: 20% fecal slurry (1:10 in PBS), 100 µM choline, and 12 h fermentation. Additionally, 10 mM of 3,3-dimethyl-1-butanol (DMB) was defined as a positive TMA production inhibitor, achieving a ~50% reduction in TMA production. Gallic acid and chlorogenic acid reported higher TMA inhibitory potential (maximum of 80–90% TMA production inhibition), with IC_50_ around 5 mM. Neither DMB nor gallic acid or chlorogenic acid reduced TMA production through cytotoxic effects, indicating mechanisms such as altered TMA-lyase activity or expression.

## 1. Introduction

The World Health Organization (WHO) reports that cardiovascular disease (CVD) is the leading cause of death worldwide (31% of all deaths), with an estimate of 17.3 million deaths a year [1]. Atherosclerosis is a relevant factor contributing to CVD development [2], and several factors can contribute to its development (i.e., endothelial dysfunction) [3]. Thus, strategies to prevent atherosclerosis and CVD could be greatly beneficial for the general population. Recently, the gut microbiota-derived metabolite trimethylamine N-oxide (TMAO) has emerged as a pro-atherogenic molecule associated with higher cardiovascular risk [4,5,6].

TMAO is formed due to consecutive gut bacterial and host metabolism of substrates such as choline and L-carnitine. The first metabolic step occurs in the gut, where bacteria oxidize these substrates into trimethylamine (TMA) through TMA-lyase enzymes. Specifically, TMA is formed from choline mainly through CutC/D enzyme complex activity [7], and from L-carnitine through CntA/B [8]. Following absorption into circulation, the second step occurs in the liver, where TMA is further oxidized by flavin-containing monooxygenase 3 (FMO3) into TMAO [9]. Various strategies have been proposed to control TMAO formation as a means to reduce CVD risk [10]. Reducing TMAO formation through TMA-lyase inhibition has shown promising anti-atherosclerotic effects in mice [11]. Another strategy would be to reduce the expression of the CutC/D or CntA/B enzyme complexes by altering the composition of the gut microbiota. Thus, the development of new strategies that target TMA-lyase(s) could be useful to manage and/or reduce TMAO formation, atherosclerosis and CVD development. 

It is believed that the reported cardioprotective effects of fruit and vegetal consumption are attributed, at least to some extent, to their bioactive phytochemicals [12,13]. While various mechanisms of action have been proposed, an emerging theory is that phytochemicals may also mediate their cardioprotective effects in part through the inhibition of TMAO formation [10]. Some intervention strategies based on phytochemical supplementation (i.e., allicin and 3,3-dimethyl-1-butanol) have been successful to reduce TMA and/or TMAO formation in animal models [11,14,15] and humans [16,17]. High-throughput inhibitor screening studies have demonstrated their utility for quickly identifying synthetic CutC/D inhibitors [18], and these types of approaches could be used to narrow down a vast array of hundreds or thousands of potential phytochemicals to a few promising candidate compounds with inhibitory activity to be subsequently tested in vivo. In this sense, Heng et al. have recently proposed a methodology to identify TMA-lyase-containing bacteria, isolate them, and use them to screen for potential compounds that inhibit choline metabolism to TMA in silico and in vitro [19]. In their approach, in silico docking studies suggested a few phytochemicals as potential TMA-lyase inhibitors, but the *in silico* approach alone does not take into account the diversity of bacteria in the gut (i.e., interaction between bacteria). Additionally, no cytotoxic tests are reported, which poses the question of whether the results are due to a TMA-lyase inhibitory effect of phytochemicals or a cytotoxic one. Thus, there still exists a lack of in vitro studies that screen the potential of phytochemicals as TMAO-reducing agents, and phytochemicals are currently being tested in vivo one at a time in a non-systematic fashion. To date, Bresciani et al. [20] are the only authors that have screened the TMA-reducing properties of several phytochemicals in vitro. Specifically, their study showed that some phenolic compounds from orange juice were able to modulate choline and L-carnitine microbial conversion to TMA in a fecal fermentation model. Of note, in vitro studies that target the gut microbiome profile and/or function can potentially provide a reasonable estimation of the activity of tested conditions in vivo, as no absorption of the tested compound is required [10]. A high-throughput in vitro screening assay could significantly advance pre-clinical data on the use of phytochemicals for reducing TMA production in a rapid, systematic, and inexpensive manner.

Thus, the aims of this study were to optimize the experimental conditions for a high-throughput, low-volume fecal fermentation method capable of screening the TMA-lowering potential of phytochemicals, and demonstrate the efficacy of selected test compounds. Optimized conditions included fecal slurry to growth media ratio, fermentation time, choline concentration, positive TMA-lyase inhibitor (DMB) concentration, and phytochemical (gallic acid and chlorogenic acid) range of concentrations.

## 2. Materials and Methods 

### 2.1. Chemicals and Reagents

Glucose, peptone, yeast extract, KCl, NaCl, Na_2_HPO_4_, KH_2_PO_4_, MgSO_4_ × 7H_2_O, CaCl_2_ × 6H_2_O, ZnSO_4_ × 7H_2_O, NaHCO_3_, ammonium formate, hemin, bile salts, Tween 80, vitamin K1, resazurin, L-cysteine, choline, choline-d9, L-carnitine, betaine, γ-butyrobetaine, TMAO, TMA, 3,3-dimethyl-1-butanol (DMB), gallic acid, chlorogenic acid, 3-(4,5-dimethylthiazol-2-yl)-2,5-diphenyl-tetrazolium bromide, ammonia and ethyl bromoacetate were purchased from Sigma-Aldrich/Millipore (St. Louis, MO, USA). Choline-d_9_, L-carnitine-d_9_, betaine-d_9_, TMAO-d_9_ and TMA-d_9_ (internal standards; IS) were also purchased from Sigma-Aldrich. Acetonitrile and water (LC-MS grade) as well as dimethyl sulfoxide (DMSO; reagent grade) were purchased from VWR International (Suwanee, GA, USA). Fecal samples from two different healthy donors (ID: 0105-0003-11 and 0128-0001-01) were obtained from OpenBiome (Cambridge, MA, USA). OpenBiome collects samples from rigorously screened donors, for which the health histories, clinical data, pathogen screen results and 16S rDNA sequences are available. Samples are processed in sterile 12.5% glycerol and 0.9% saline buffer at 2.5 mL of buffer per gram of stool, and filtered through a 330 µm filter to remove large particulate matter, and frozen at −80 °C until use.

### 2.2. Culture Media Preparation

Fermentation media composition and preparation were adapted from Alqurashi et al. [21]. The composition of 1 L of growth medium was 2 g peptone water, 2 g yeast extract, 0.1 g NaCl, 40 mg Na_2_HPO_4_, 40 mg KH_2_PO_4_, 10 mg MgSO_4_·7H_2_O, 10 mg CaCl_2_·6H_2_O, 2 g NaHCO_3_, 50 mg hemin, 0.5 g bile salts, 2 mL Tween 80, 10 µL vitamin K1, 1 mg resazurin, and 0.5 g L-cysteine. Two different 500 mL solutions were prepared at 2X concentration. Solution A included all components except for resazurin and L-cysteine, which were included in Solution B. Both solutions were filter-sterilized separately through a 0.22-µm sterile filtering system (Corning, Corning, NY, USA), and pH was adjusted to 6.8. Solution B was then boiled for 10 min. Thereafter, both solutions were sparged overnight (minimum of 8 h) with N_2_ (g) under agitation and were then combined in the anaerobic chamber (O_2_ < 15 ppm) to 1X concentration. This growth media mixture was used to grow bacteria in fecal slurries under different experimental conditions. The PBS 1X solution was filter-sterilized (22 µm sterile filtering system, Corning, Corning, NY, USA) and sparged overnight with N_2_ to maintain sterile and anaerobic working conditions. Fecal samples were thawed, pooled (1 mL each from2 different donors) and diluted 10X with PBS immediately prior to use. This is referred to as “fecal slurry”. 

### 2.3. Anaerobic Chamber Conditions

All fermentations were carried out inside a 4-glove 855-ACB anaerobic chamber from Plas-Labs (Lansing, MI, USA). The anaerobic chamber was filled with a mixed gas composed of 5% H_2_, 5% CO_2_ and 90% N_2_ provided by Airgas (Durham, NC, USA). H_2_ (typically 2–3%) and O_2_ (typically < 15 ppm) levels were monitored with a CAM-12 anaerobic monitor from Coy Lab Products (Grass Lake, MI, USA). Temperature was set at 37 °C, and it was maintained constant (recorded values within 36–38 °C) throughout the fermentation procedure. Humidity ranged between 40 and 50% during all experiments.

### 2.4. Fermentation Conditions

#### 2.4.1. Optimization of Choline and Fecal Slurry Conditions

Choline concentration (5–100 µM) and fecal slurry percentage (5–45%) in the final fermentation mixtures with media were studied in various combinations to select the optimal fermentation conditions. Range selection was based on the study of Bresciani et al. [20]. In 1.1 mL 96-well plates, 405 µL of growth media and 90 µL of choline stock solution (1000–50 µM) in PBS 1X were mixed with fecal slurry (45–405 µL) to reach either 5, 10, 20 or 45% of fecal slurry concentration and 0, 5, 10, 25, 50 or 100 µM concentrations of choline, in various combinations. Each well was brought to a final volume of 900 µL with PBS 1X. All solutions were pre-heated at 37 °C. At 0, 1, 2, 3, 4, 5, 6, 8, 10, 12, 16, 20, 24, 30 and 36 h after fecal slurry inoculation, 50 µL of sample were collected. Immediately after collection, 50 µL of acetonitrile were added to samples, and those were immediately frozen at −80 °C until use for TMA-related compound extraction. Optical density at 600 nm was measured for samples at 36 h (*n* = 3). 

#### 2.4.2. Optimization of 3,3-Dimethyl-1-butanol Concentration

In order to validate the results of our study, the known TMA-lyase inhibitor DMB was used as a positive control [11]. DMB concentrations (0.2–10 mM) were studied to select an optimal TMA-lyase inhibitor concentration, as this range has been reported to inhibit choline conversion to TMA in vitro and in vivo [11]. Fermentation conditions were the same as above, with the exception of choline and fecal slurry concentrations, which were, respectively fixed at 100 µM and 20% based on optimization results. Samples were drawn at 0, 4, 6, 8, 10, 12, 16 and 24 h after fecal slurry inoculation, and were treated as previously explained. Optical density was measured at 600 nm for samples at 24 h (*n* = 6).

#### 2.4.3. Screening for the Inhibitory Effect of Gallic Acid and Chlorogenic Acid 

In order to evaluate the inhibitory effect of two representative phytochemicals (gallic acid and chlorogenic acid) on microbial oxidation of choline to TMA, these two compounds were tested in optimal fermentation conditions (choline 100 µM and fecal slurry 20%) in a concentration range within 0.1–10 mM (concentrations chosen for comparison with DMB). A choline-free treatment and a DMB (10 mM) treatment were included as controls. A volume of 50 µL of sample was drawn at 0, 8 and 12 h, and the optical density was read at 600 nm (*n* = 6). Then, samples were treated as in previous steps to quantify TMA-related compounds. 

### 2.5. Extraction and Analysis of TMA-Related Compounds

Choline, L-carnitine, betaine, γ-butyrobetaine, TMAO and TMA were extracted according to the previously reported methodology [22,23]. TMA required a derivatization process to the quaternary amine compound ethyl betaine in order to facilitate LC-MS/MS ionization. Briefly, 25 µL of sample were mixed with 20 µL of TMA-d_9_ internal standard solution (0.25 µM, for derivatization to ethyl betaine-d_9_), 8 µL concentrated ammonia and 120 µL of ethyl bromoacetate (20 mg/mL), and left to sit for 30 min. Then, 120 µL of 50% acetonitrile/0.025% formic acid in distilled water were added. TMA samples were filtered through AcroprepAdv 0.2 µm WWPTFE 96-well filtering plates (Pall Corporation, Port Washington, NY, USA) by centrifugation (10 min, 3400× *g*), collected in a new 96-well collection plate and frozen at −80 °C until UHPLC-MS/MS analysis. No more than 48 h passed between TMA/choline extraction and their analysis. To extract the other compounds, 25 µL of sample were mixed with 10 µL of ZnSO_4_ (5% *w*/*v* in water) and 100 µL of a mixture of internal standard compounds (choline-d_9_, L-carnitine-d_9_, betaine-d_9_, and TMAO-d_9_) at a concentration of 0.25 µM (except 1.20 µM for L-carnitine-d_9_) in 96-well plates. After sonication for 5 min in a water bath, samples were filtered and stored as described above. 

### 2.6. Chromatographic Analysis of TMA-Related Compounds

TMA was analyzed separately from the other compounds, but with the same UHPLC-ESI-MS/MS method. Briefly, compound separation was achieved on a Waters Acquity UPLC system (Milford, MA, USA) with an ACQUITY BEH HILIC column (1.7 µm, 2.1 × 100 mm) coupled to an ACQUITY BEH HILIC pre-column (1.7 µm, 2.1 × 5 mm) (Waters). Mobile phases consisted of 15 mM ammonium formate in water (pH 3.5) (A) and acetonitrile (B). The gradient was set to isocratic at 80% B for 3 min, with a flow rate of 0.65 mL/min. Column temperature was set at 30 °C, and autosampler at 10 °C. Quantification was achieved by coupling the above system with a Waters Acquity triple quadrupole mass spectrometer. Source and capillary temperatures were set at 150 and 400 °C, respectively. Capillary voltage was set at 0.60 kV, and desolvation and cone gas flow (both N_2_) were set at 800 and 20 L/h, respectively. Electrospray ionization (ESI) was operated in the positive mode, and data were acquired using the multiple reaction monitoring (MRM) mode. Multi-reaction monitoring (MRM) fragmentation conditions of choline, L-carnitine, betaine, γ-butyrobetaine, TMAO and TMA, as well as IS compounds can be found in Table S1.

### 2.7. TMA-Related Compounds Sample Quantification

For sample quantification, 45% growth media in PBS 1X was spiked with 7 different concentrations each of choline, L-carnitine, betaine, γ-butyrobetaine, TMAO and TMA standards to obtain external calibration curves in a relevant background matrix. Samples were quantified by interpolating the analyte/analyte-d_9_ (IS) peak abundance ratios in the standard curves (betaine-d_9_ was used as the standard for γ-butyrobetaine, due to the lack of availability of a deuterated γ-butyrobetaine standard). Data acquisition was carried out using Masslynx software (V4.1 version, Waters). For choline, the concentration of choline at each time point found in the 0 µM choline treatment in each fecal slurry percentage (5–45%) from the experimental samples was subtracted from the same time point and same fecal slurry percentage, to account for background choline in the fecal samples. For TMA, the concentration of TMA reported at 0 h from 0 µM choline in each fecal slurry percentage (5–45%) from the experimental samples was subtracted from any other time point with the same fecal slurry concentration to account for background TMA in the fecal samples at baseline. Method sensitivity was determined by limit of detection (LOD) and limit of quantification (LOQ), respectively defined as the concentration of analyte corresponding to 3 and 10 times the signal/noise ratio. Method detection (MDL) and quantification (MQL) limits were calculated for the analysis of 25 µL of non-diluted fecal fermentation media samples. Method quality parameters can be found in Table S2.

To determine the background (endogenous) levels of TMA and related compounds in experimental conditions, fecal slurry 1:10 in PBS 1X and fermentation media (45% growth media + 55% PBS 1X) with fecal slurry (1:10 in PBS 1X) at a final concentration of 20% were analyzed as previously stated. Given the presence of TMA-related compounds in the background, these samples were quantified using water to construct the calibration curves. 

### 2.8. Cell Count

The number of cells present in the fermentation media was approximated by reading the optical density at 600 nm in a SpectraMax iD3 plate reader (Molecular Devices, San Jose, CA, USA). Cell count was performed in 100 µL of sample drawn at the last timepoint of the fermentation (12–36 h), which depended on experimental conditions. Results are expressed as percentages of change versus choline-free conditions ± SEM (*n* = 3 or 6).

### 2.9. Mitochondrial Toxicity Test

A mitochondrial toxicity test (MTT) was used to estimate cell viability. Briefly, 10 µL of fermentation mixture was mixed with 80 µL of pre-heated (37 °C) PBS 1X with glucose 0.2% (*m*/*v*) and 10 µL of 3-(4,5-dimethylthiazol-2-yl)-2,5-diphenyl-tetrazolium bromide at a concentration of 25 mg/mL in PBS 1X [24]. Samples were allowed to react for 30 min under anaerobic conditions (37 °C and O_2_ < 15 ppm), and the resulting formazan crystals were resuspended to a final volume of 1 mL with DMSO by stirring for 30 min. An aliquot of 100 µL was read at 560 nm in a SpectraMax iD3 plate reader (Molecular Devices, San Jose, CA, USA). All reagents were prepared with overnight-sparged PBS 1X and filter-sterilized 22 µm sterile filtering system, Corning) before their use. An MTT test was performed at the last timepoint of the fermentation (12–24 h), which depended on experimental conditions. Results are expressed as percentages of change versus choline-free conditions ± SEM (*n* = 3 or 6). 

### 2.10. Statistics

Prism 8.0 (GraphPad, La Jolla, CA, USA) was used for statistical analyses and graph creation purposes. Two-way ANOVA (main effects: dose of choline or fecal slurry, and time) was used to estimate differences in choline and TMA kinetic curves (if significant main dose effects were detected, Tukey post hoc tests were employed to determine differences in dose means between groups). One-way ANOVA (Tukey post hoc test) or Student’s *t*-test were used for any other purpose. For all ANOVA procedures, post hoc tests were only applied if an overall significant main effect (2-way) or overall treatment effect (1-way) were first detected. In all cases, statistical significance was stablished a priori as *p* < 0.05. IC_50_ for gallic acid and chlorogenic acid were calculated using a 4-factor sigmoidal fitting with interpolation of concentrations producing a 50% of maximum TMA levels (mean TMA of *n* = 6 levels were used). 

## 3. Results

### 3.1. Background Concentrations of TMA-Related Compounds

To monitor the presence of endogenous TMA and TMA-containing substrates, TMA, choline, L-carnitine, betaine, γ-butyrobetaine and TMAO levels were quantified in fecal slurry 1:10 in PBS 1X and a 20% dilution of this mixture in fermentation background (representing everything added to fermentation experiments except exogenous choline substrate: 45% growth media + 55% PBS 1X) (Table 1). Due to the presence of these compounds in fermentation media, calibration curves were prepared in water (whereas calibration curves for experimental fermentations were prepared using the background matrix). Endogenous TMA was not detected in either the fecal slurry or the fermentation background. However, our analysis indicates that the original fecal slurry contains relevant concentrations of TMA-producing substrates: choline (~80 µM) and betaine (~250 µM). Although the original fecal slurry is 1:10 diluted in PBS 1X, and fermentations include concentrations of fecal slurry 1:10 between 5 and 45% (concentration of the original fecal slurry between 0.5 and 4.5%), fermentation background (45% growth media + 55% PBS 1X) inoculated with fecal slurry 1:10 to a final proportion of 20% contains even higher concentrations of choline (~225 µM) and betaine (~1120 µM). The contribution of these endogenous substrates to TMA production in the assay must, therefore, be taken into account.

### 3.2. Optimization of Choline and Fecal Slurry Concentrations

**Figure 1:**
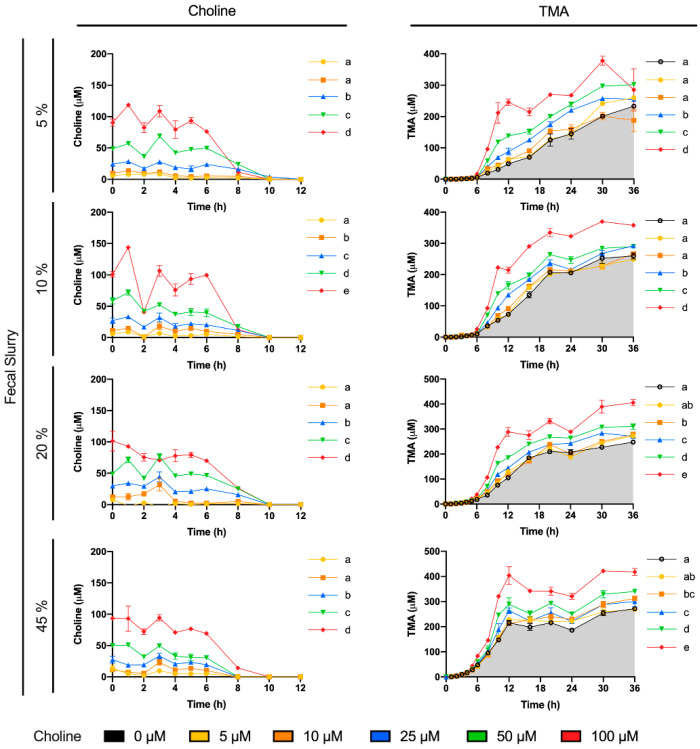
Choline (**left**) and TMA (**right**) kinetic curves obtained by fermenting exogenous choline (0–100 µM) with different percentages of fecal slurry (5–45% *v*/*v*). For choline curves, data beyond 12 h are not shown for clarity (concentrations were all in the non-detected or non-quantified range), and time-matched choline concentrations in the background (from the mean detected choline levels in the 0 µM choline treatment) are subtracted from choline-supplemented conditions in order to reflect only exogenous choline levels. Results are expressed as mean (µM choline or TMA) ± SEM (*n* = 3). Different letters indicate statistical differences (*p* < 0.05) in main effect of choline dose or fecal slurry percentage by two-way ANOVA (Tukey’s post hoc test). Factors for two-way ANOVA were choline concentration or fecal slurry proportion and time. Grey shading indicates background TMA production.

**Figure 2:**
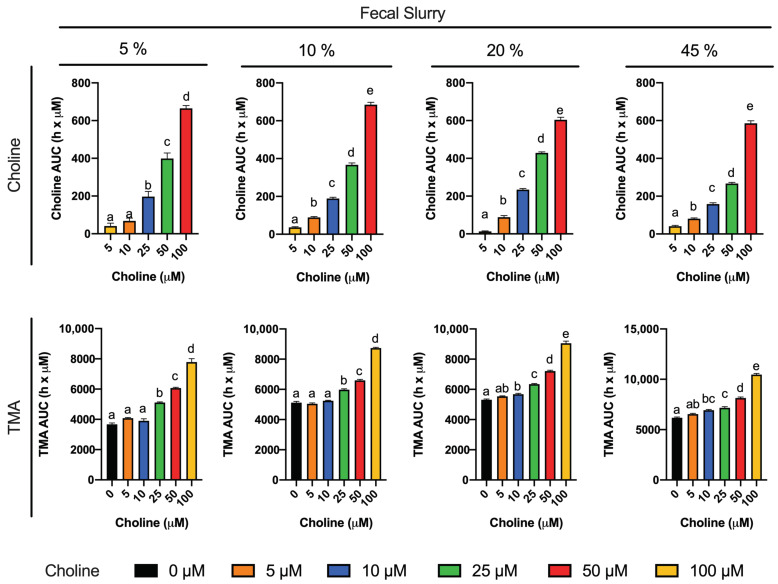
Areas under the curve (AUC) of choline (**top**) and TMA (**bottom**) kinetic curves obtained by fermenting choline (0–100 µM) with different percentages of fecal slurry (5–45%). For choline AUCs, time-matched choline concentrations in the background (0 µM choline) are subtracted from choline-supplemented conditions (values for 0 µM choline AUCs are thus 0, and not shown). Results are expressed as mean (h x µM of choline or TMA) ± SEM (*n* = 3). Different letters indicate statistical differences (*p* < 0.05) by one-way ANOVA (Tukey’s post hoc test). TMA levels at 0 µM choline were not detected, and thus, have not been included for clarity.

Choline consumption and TMA formation were monitored in fermentations with 5–45% of fecal slurry (1:10 in PBS) and exogenous choline concentrations between 0 and 100 µM (above background) in various combinations (Figure 1). Regardless of initial choline concentration or fecal slurry percentage, choline concentrations remained somewhat constant up to 6 h, after which time they started to decrease until reaching non-detected levels (above the background) at 10–12 h for all treatments. At the low fecal slurry concentrations of 5 and 10%, the addition of the lowest choline doses of 5 and 10 µM resulted in a TMA kinetic curve statistically equal to the 0 µM choline treatment (no choline added, background choline only). This was also true for the curve of choline 5 µM at 20 and 45% fecal slurry. TMA kinetic curves arising from exogenous initial choline concentrations ≥ 25 µM were statistically different from the background, as well as each other, in all fecal slurry concentrations. The statistical trends in choline and TMA kinetic curves were also conserved in the analysis of their AUCs (Figure 2). TMA production in the background fermentations (0 µM exogenous choline added) reached ~250 µM, likely due to the endogenous substrates discussed above. TMA production reached a maximum level of ~400 µM (for choline 100 µM), or ~150 µM above background. A plateau in TMA production was achieved at 12 h in fecal slurry 45%, at 16 h in fecal slurry 20%, at 20 h in FS 10%, and was not reached at fecal slurry 5%. Overall, final TMA concentrations were similar regardless of fecal slurry concentrations.

### 3.3. Assessment of Spontaneous Oxidation of Choline and Background TMA Contribution

**Figure 3:**
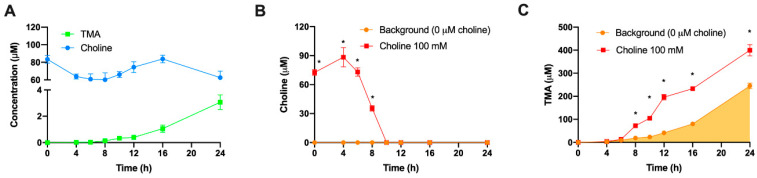
Choline and TMA changes in fecal slurry-free fermentation conditions (**A**). Changes in choline (**B**) and TMA (**C**) concentrations in fermentations with fecal slurry 20% and ±100 µM choline. In (**B**), time-matched choline concentrations in the background (from the mean detected choline levels in the 0 µM choline treatment) are subtracted from choline-supplemented conditions in order to reflect only exogenous choline levels. Results are expressed as mean (µM choline or TMA) ± SEM (*n* = 6). * indicates statistical differences (*p* < 0.05) by time-matched two-way ANOVA (Tukey’s post hoc test). Orange shading indicates background TMA production.

Several controls to validate the utility of this model were employed. First, we wished to verify that the observed conversion of exogenous choline and production of TMA was primarily due to bacterial metabolic activity, as opposed to spontaneous degradation. In microbiota-free (PBS 1X only, instead of fecal slurry 1:10) and anerobic conditions (Figure 3A), choline concentrations remained > 70% of initial concentration (100 µM) over 24 h, and spontaneous TMA production barely occurred (reaching a maximum of ~3 µM at 24 h). This was in stark contrast to the loss of ~100% choline, and production of TMA at ~250–350 μM, observed at 24 h when microbiota was present from the fecal slurry (Figure 1). This provides strong evidence that a microbial factor was responsible for the majority of choline conversion to TMA. Next, we wished to ascertain the contribution of the microbial background, without choline supplementation, to TMA production. We thus compared TMA production with and without exogenous choline (100 µM). In choline-supplemented fermentations (Figure 3B,C), choline levels started to decrease at 6 h, and became not detected at 10, while TMA levels reached a 24 h concentration of ~400 µM. Although choline-free (no exogenous choline added) conditions did not result in detectable choline (above background) at any timepoint, TMA concentrations in these samples still reached ~245 µM at 24 h, suggesting significant contribution of the background TMA precursors (endogenous choline and betaine, etc.) to TMA production in the assay. However, there was significant separation in TMA production between 0 and 100 µM exogenous choline treatments, allowing for use of this assay despite significant background TMA production.

### 3.4. Optimal DMB Concentration

**Figure 4:**
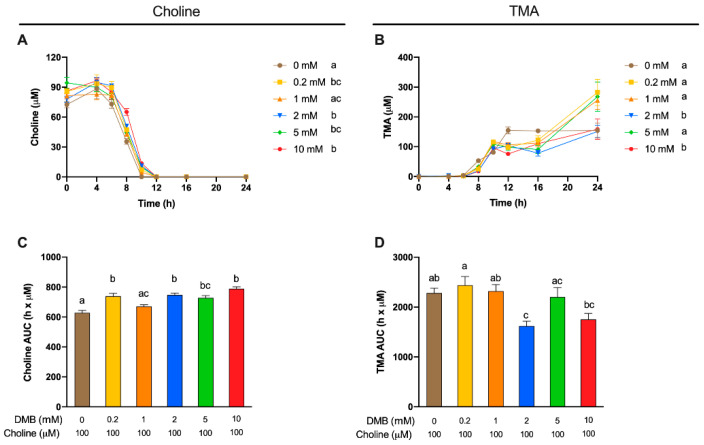
Choline (**A**) and TMA (**B**) kinetic curves obtained from fermentations with fecal slurry 20%, choline 100 µM and DMB concentrations of 0–10 mM. Results are expressed as mean (µM choline or TMA) ± SEM (*n* = 6), and for choline, time-matched choline concentrations in the background (0 µM choline) are subtracted from choline-supplemented conditions. Areas under the curve (AUCs) from choline (**C**) and TMA (**D**) fermentations. Results are expressed as mean (h × µM of choline or TMA) ± SEM (*n* = 6), and for choline, time-matched choline concentrations in the background (0 µM choline) are subtracted from choline-supplemented conditions. Different letters indicate statistical differences (*p* < 0.05) by two-way ((**A**,**B**) main effect of DMB concentration) or one-way (**C**,**D**) ANOVA (Tukey’s post hoc test). Factors for two-way ANOVA were DMB concentration and time.

A second set of experiments was then carried out to study the best DMB concentration (0.2–10 mM) to inhibit TMA production (Figure 4), in order to select a positive control concentration for subsequent experiments. Fermentations with DMB were carried out with fecal slurry 20% and choline concentrations of 100 µM, based on the optimization studies described above. The supplementation with DMB produced statistically different choline kinetic curves compared with DMB-free conditions in all cases except for DMB 1 mM, indicating DMB inhibition of TMA production. At metabolically relevant timepoints (≥6 h), choline kinetic curves of DMB-supplemented conditions were above the one from DMB-free (0 mM) conditions, indicating preservation of exogenous choline. Of note, choline reached non-detectable levels (above background) at 10 h in DMB-free conditions and at 12 h in DMB-supplemented conditions, indicative of a lag in choline utilization due to DMB. While DMB-free conditions reached a plateau in TMA formation at 12 h, this was not true for DMB-treated conditions (Figure 4B). Interestingly, 2 and 10 mM DMB decreased TMA levels from 12 to 24 to reach similar levels as DMB-free levels, whereas concentrations of DMB of 0.2, 1 and 5 mM reported a higher TMA production at 24 h. Overall, the kinetic curves of TMA formation and the AUC of those (Figure 4C,D) reported few statistical differences between groups. The DMB 10 mM kinetic curve was different from that of the 0 mM DMB, although AUCs are not statistically different. Surprisingly, DMB 2 mM, but not DMB 5 mM, reports a statistically different curve and AUC compared to 0 mM DMB. Overall, based on Figure 4B,D, 2 and 10 mM DMB appeared to effectively reduce TMA production.

**Figure 5:**
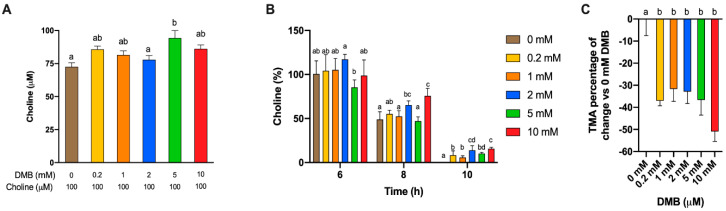
Initial choline concentrations at fermentation with fecal slurry 20%, choline 100 µM and DMB concentrations of 0–10 mM (**A**). Relative changes in choline at 6, 8 and 10 h against initial choline concentrations (**B**). Relative changes in TMA concentration at 12 h against DMB 0 mM (**C**). Results are expressed as mean (µM or%) ± SEM (*n* = 6). Different letters indicate statistical differences (*p* < 0.05) by one-way ANOVA (Tukey’s post hoc test) within each time point.

Apparent choline concentrations at initial conditions (0 h) were different between DMB-free and DMB 5 mM (Figure 5A). Choline concentrations for all timepoints were relativized to initial choline concentrations and subjected to statistical analysis (Figure 5B). At 6 h, only 2 mM DMB reported higher percentages of choline than DMB-free conditions, while at 8 h, concentrations of 2 and 10 mM DMB presented higher percentages of choline. At 10 h, the last time point at which choline was quantified in DMB-treated conditions, all groups presented higher relative choline percentages than the DMB-free condition (not quantified). TMA concentrations were relativized to TMA concentrations in 0 mM DMB (Figure 5C). No statistical differences in TMA reductions were reported between DMB dose >0 mM (due to large variability). All concentrations reached a >30% reduction in TMA concentration, the highest being a reduction of 50.9 ± 4.50% for DMB 10 mM. These results indicate that DMB preserves choline and inhibits TMA production at the range of concentrations tested.

**Figure 6:**
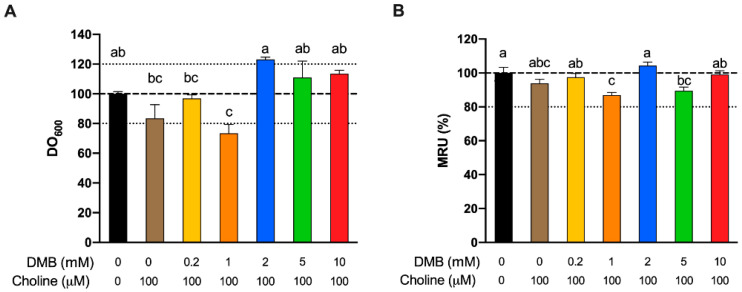
Relative approximate cell count (600 nm optical density) fermentations at 24 h as a % of DMB-free choline-free conditions (**A**). Relative changes in mitochondrial respiration units (MRU) at 24 h as a % of DMB-free choline-free conditions (**B**). All fermentations were carried out with fecal slurry 20%. Results are expressed as mean (%) ± SEM (*n* = 6). Different letters indicate statistical differences (*p* < 0.05) by one-way ANOVA (Tukey’s post hoc test).

We next sought to ascertain whether inhibition of TMA formation by DMB was due to reduced bacterial cell numbers (due to cell death or inhibition of cell growth), or by another mechanism. Cell number, measured as optical density at 600 nm, and mitochondrial respiration (MTT) were used to assess any toxic or growth-inhibition effect of DMB treatment at 24 h (Figure 6A,B, respectively). No differences were reported between background (choline and DMB-free conditions) and DMB-free conditions + 100 µM choline. Only DMB 1 mM reported a significantly lower number of cells than the background, and this was >20% change. DMB 1 mM was also significantly different than DMB-free conditions and DMB ≥ 2 mM. DMB 5 and 10 mM were not significantly different from DMB-free conditions. DMB-free mitochondrial activity (MTT) was not statistically different from any of the DMB-administered groups, or the background for that matter. The only groups that presented a lower mitochondrial activity than the background were DMB 2 and 5 mM, but these differences did not reach a >20% change. Overall, changes in cell number or viability did not generally explain observed reductions in TMA production.

### 3.5. Effect of Gallic Acid and Chlorogenic Acid on Choline Metabolism and TMA Production

**Figure 7:**
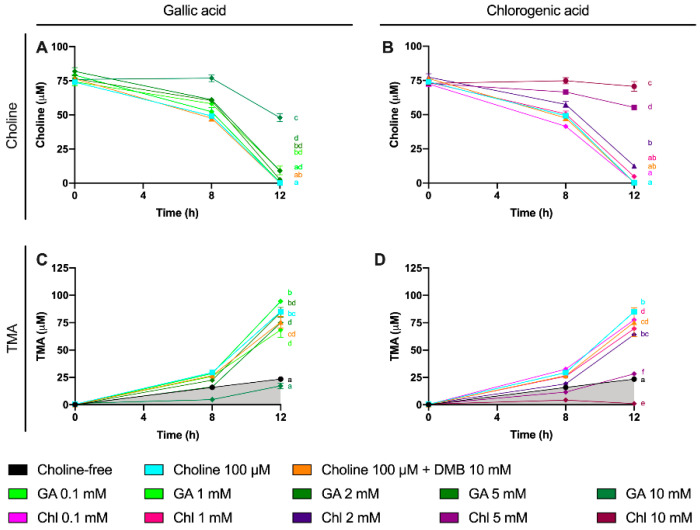
Choline (**A**,**B**) and TMA (**C**,**D**) kinetic curves obtained from optimal fermentations conditions (fecal slurry 20% and choline 100 µM) supplemented with gallic acid or chlorogenic acid (0.1–10 mM). Results are expressed as mean (µM choline or TMA) ± SEM (*n* = 6), and for choline, time-matched choline concentrations in the background (0 µM choline) are subtracted from choline-supplemented conditions. Different letters indicate statistical differences (*p* < 0.05) by two-way (main effect of gallic acid or chlorogenic acid dose) ANOVA (Tukey’s post hoc test). Factors for two-way ANOVA were gallic acid or chlorogenic acid concentration and time. Grey shading indicates background TMA production. Abbreviations: TMA, trimethylamine; GA, gallic acid; and Chl, chlorogenic acid.

**Figure 8:**
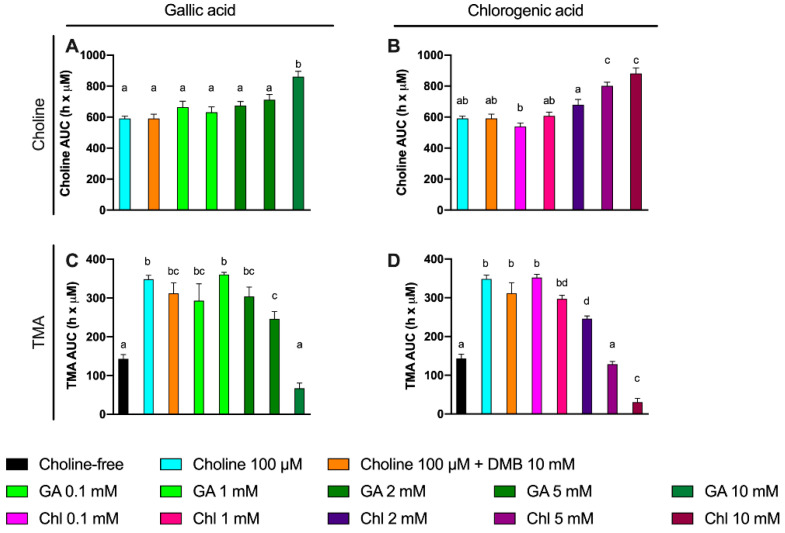
Area under the curve (AUC) of choline (**A**,**B**) and TMA (**C**,**D**) kinetic curves obtained at optimal fermenting conditions (fecal slurry 20% and choline 100 µM) supplemented with gallic acid or chlorogenic acid (0.1–10 mM). For choline AUCs, time-matched choline concentrations in the background (0 µM choline) are subtracted from choline-supplemented conditions. Results are expressed as mean (h × µM of choline or TMA) ± SEM (*n* = 6). Different letters indicate statistical differences (*p* < 0.05) by one-way ANOVA (Tukey’s post hoc test). Abbreviations: TMA, trimethylamine; GA, gallic acid; and Chl, chlorogenic acid. TMA levels at 0 µM choline were not detected, and thus, have not been included for clarity.

A third set of experiments was then conducted to assess the inhibition potential of two representative phenolic compounds that are abundant in the human diet (gallic acid and chlorogenic acid) on the microbial conversion of choline to TMA (Figure 7). Fermentations were carried out with previously optimized conditions (fecal slurry 20% and choline concentrations of 100 µM), and a DMB 10 mM condition was included as a positive control (known TMA-lyase inhibitor) based on the optimization described above. In gallic acid-supplemented conditions, choline kinetic curves were statistically lower from 100 µM choline alone at ≥2 mM gallic acid (Figure 7A), and AUCs were different only at 10 mM (Figure 8A). TMA kinetic curves were significantly lower from 100 µM choline only at ≥5 mM gallic acid (Figure 7C), and so were AUCs (Figure 8C). In chlorogenic acid-supplemented conditions, choline kinetic curves were statistically lower from 100 µM choline only at ≥2 mM chlorogenic acid (Figure 7B), and AUCs were significantly higher at ≥5 mM chlorogenic acid (Figure 8B). TMA kinetic curves were significantly lower from 100 µM choline only at ≥5 mM chlorogenic acid (Figure 7D), and AUCs were significantly lower from 100 µM choline at ≥2 mM chlorogenic acid (Figure 8D). Of note, gallic acid 10 mM and chlorogenic acid 5 mM and 10 mM reported TMA production rates equal or lower than choline-free (0 µM) conditions, which highlights their high capacity to reduce TMA formation (i.e., they reduced even background TMA formation).

**Figure 9:**
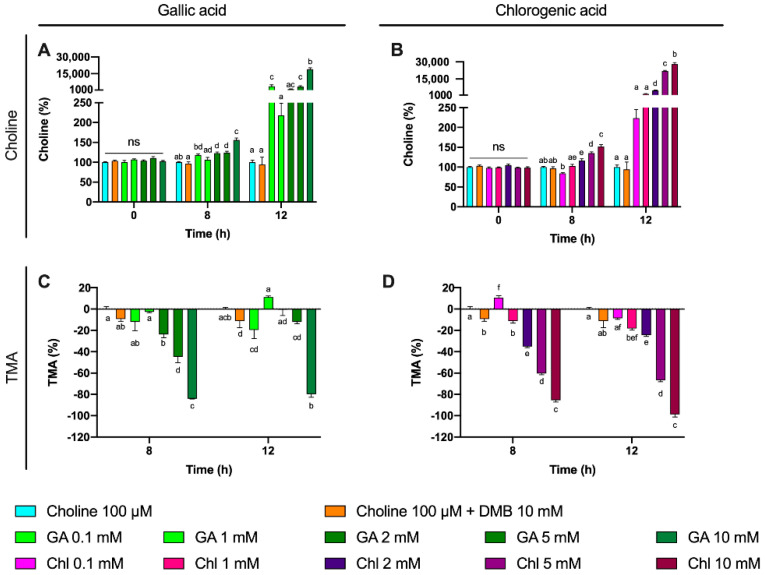
Gallic acid and chlorogenic acid (0–10 mM) relative choline percentage increase effect against choline-supplemented (100 µM) conditions (**A**,**B**). Gallic acid and chlorogenic acid (0.1–10 mM) relative TMA percentage decrease effect against choline-supplemented (100 µM) conditions (**C**,**D**). All fermentations were carried out at optimal conditions (fecal slurry 20% and choline 100 µM). Results are expressed as mean (%) ± SEM (*n* = 6). Different letters indicate statistical differences (*p* < 0.05) by one-way ANOVA (Tukey’s post hoc test). Abbreviations: TMA, trimethylamine; GA, gallic acid; and Chl, chlorogenic acid.

We then evaluated the % inhibition of gallic acid and chlorogenic acid in choline use and TMA production out to 12 h (Figure 9). No changes in choline concentrations relative to choline 100 µM were reported at time 0 h for gallic acid or chlorogenic acid. However, the relative choline levels were higher at gallic acid ≥ 2 mM at 8 and 12 h. This was also true for gallic acid 0.1 mM at 12 h, but not for gallic acid 1 mM at any timepoint. TMA relative percentage versus 100 µM choline was statistically lower than 100 µM choline at gallic acid concentrations ≥ 2 mM at 8 h, and at 10 mM at 12 h. Inhibition of ~80% of TMA formation at 8 and 12 h was achieved by the highest dose of gallic acid. Chlorogenic acid reported higher relative choline concentrations at ≥2 mM than 100 µM choline both at 8 and 12 h. TMA relative changes were statistically different from 100 µM choline at ≥1 mM at 8 h and 12 h. The effects on choline relative changes of gallic acid and chlorogenic acid were more relevant at 12 h, reporting > 1000% increases in remaining choline concentrations in some conditions (5 and 10 mM), while the % TMA reductions were relatively constant at 8 and 12 h, achieving inhibition of ~80% of TMA formation at 8 and 12 h by the highest dose of chlorogenic acid.

**Figure 10:**
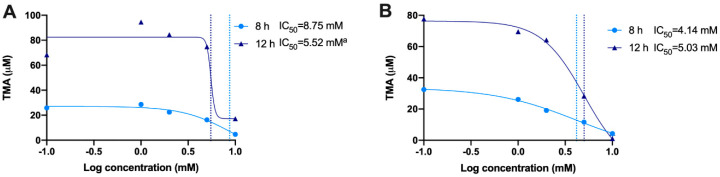
Calculation of IC_50_ for gallic acid (**A**) and chlorogenic acid (**B**) at 8 and 12 h. IC_50s_ were calculated using a 4-factor sigmoidal fitting with interpolation of concentrations producing 50% of maximum TMA levels (mean TMA production from *n* = 6 replicates was used). ^a^ Ambiguous IC_50_.

We then calculated the IC_50_ for gallic acid and chlorogenic acid both at 8 and 12 h by analyzing the levels of TMA production (Figure 10). Although we attempted to calculate IC_50_ for gallic acid at 12 h, the results (5.52 mM) were flagged as “ambiguous” and may not be valid. IC_50_ for gallic acid at 8 h was of 8.75 mM. Chlorogenic acid reported lower IC_50_s than gallic acid, both at 8 and 12 h (4.14 and 5.03 mM, respectively).

Besides choline and TMA, we further studied betaine, L-carnitine, γ-butyrobetaine and TMAO, as these can all potentially give rise to TMA via microbial activity [8,25,26]. Out of these, only betaine was detectable in fermentation culture media with fecal slurry 20%. Due to its high peak area in the background (fecal slurry 20% in growth media and PBS 1X), its quantification would provide inaccurate values. Thus, we plotted betaine/betaine-d_9_ peak area ratio over time, and these relative from gallic acid 10 mM and 0.1 mM were statistically different from choline 100 µM, while gallic acid 1 mM, 2 mM and 5 mM were not (Figure S1A). This could be attributed to the fact that betaine/betaine-d_9_ levels increase from 0 to 8 h and then decrease at 12 h, and to the fact that while steady from 0 to 8 h, betaine/betaine-d_9_ levels in gallic acid 10 mM increase at 12 h. When these data were normalized to choline 100 µM, no changes were reported at 0 h, and only gallic acid 0.1 mM reported an increase in betaine relative levels. However, at 12 h, betaine relative levels of gallic acid 10 mM were different from the rest of the treatments, including choline 100 µM (Figure S1C). The kinetic curves of chlorogenic acid were different from choline 100 µM at all concentrations (Figure S1B). No significant changes were reported in relative betaine levels at 0 h, and all chlorogenic acid concentrations reported a higher relative betaine level at 8 h compared to choline 100 µM. This was maintained at 12 h for all chlorogenic acid concentrations but for 2 mM (Figure S1D).

**Figure 11:**
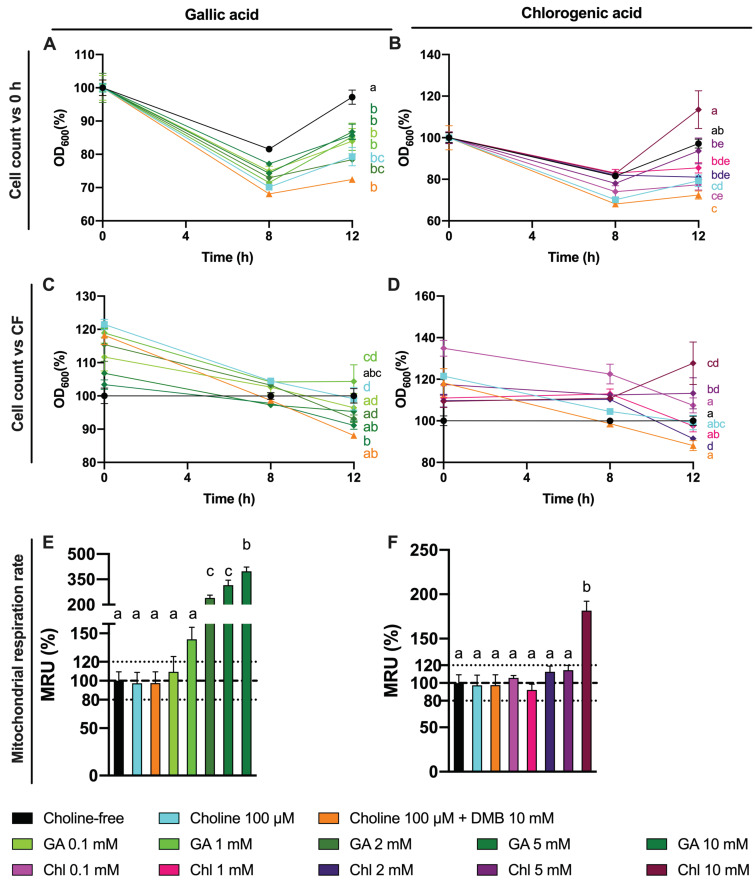
Effect of gallic acid and chlorogenic acid (0.1–10 mM) on approximate cell count (optical density at 600 nm) over time relativized to initial (0 h) absorbance values (**A**,**B**). Effect of gallic acid and chlorogenic acid (0.1–10 mM) on approximate cell count (optical density at 600 nm) relativized to choline-free (0 µM) conditions at the end (12 h) of the fermentation (**C**,**D**). Effect of gallic acid and chlorogenic acid (0.1–10 mM) on mitochondrial respiration rate relativized to choline-free (0 µM) conditions at the end (12 h) of the fermentation (**E**,**F**). All fermentations were carried out at optimal conditions (fecal slurry 20% and choline 100 µM). Results are expressed as mean (%) ± SEM (*n* = 6). Different letters indicate statistical differences (*p* < 0.05) by one-way or two-way ANOVA (Tukey’s post hoc test). Factors for two-way ANOVA were gallic acid or chlorogenic acid concentration and time. Abbreviations: DMB, 3,3-dimethyl-1-butanol; GA, gallic acid; and Chl, chlorogenic acid.

The observed changes in choline utilization and TMA production could arise from a toxic effect of gallic and chlorogenic acid. To assess this, cell number, analyzed as optical density at 600 nm, and mitochondrial respiration (MTT) were used to assess any toxic effect of gallic acid (0.1–10 mM) and chlorogenic acid (0.1–10 mM) treatments at 12 h (Figure 11). This time, we analyzed cell number over the three sampling time points (0, 8 and 12 h). Cell number curves were normalized to initial (0 h) values (Figure 11A,B). Relative changes in cell count compared to choline-free (0 µM) conditions were also analyzed by the end of the fermentation (Figure 11C,D), and so was mitochondrial respiration rate (Figure 11E,F). There were no statistical differences in the kinetics of cell growth between gallic aid treatments and choline 100 µM, and all were different and below choline-free (0 µM) conditions. At the end of the fermentation (12), gallic acid did not produce any change in relative cell count, but mitochondrial respiration rate compared to choline-free conditions was >120% at gallic acid ≥ 1 mM (statistically significant at gallic acid ≥ 2 mM, and reaching up to 350%). The curves of cell count for 5 and 10 mM chlorogenic acid were different from choline 100 µM, and all but chlorogenic acid 10 mM were different from choline-free (0 µM) conditions. At 12 h, chlorogenic acid only reached a statistical increase in relative cell count at 10 mM, which was higher than 120%. This was also true for mitochondrial respiration rate. Of note, all cell count curves, regardless of the treatment, reported a decrease in optical density at 600 nm at 8 h, and an increase at 12 which only reached values above 100% of the initial values for chlorogenic acid 10 mM.

**Figure 12:**
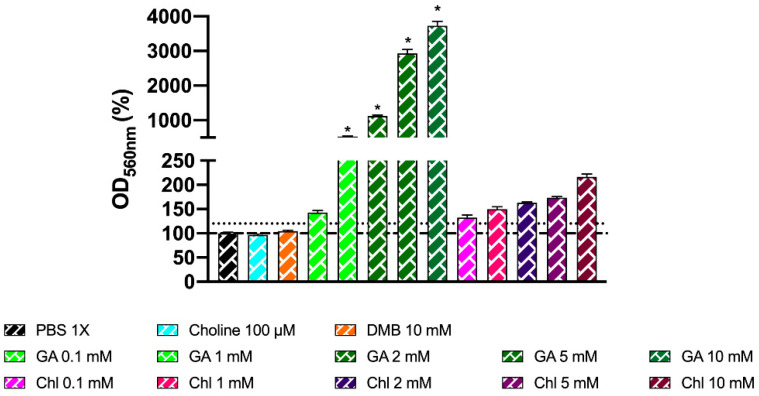
Effect of choline 100 µM, DMB 10 mM, gallic acid (0.1–10 mM), and chlorogenic acid (0.1–10 mM) on MTT reagent reduction to colored product. Reactions occurred in anaerobic conditions (O_2_ < 15 ppm) for 30 min with PBS 1X (filter-sterilized and N_2_-sparged) as diluent, without fermentation media or fecal slurry. Results are expressed as mean % compared to the vehicle conditions) ± SEM (*n* = 6). * *p* < 0.0001 by one-way ANOVA (Dunnett’s post hoc test versus PBS 1X). Abbreviations: DMB, 3,3-dimethyl-1-butanol; GA, gallic acid; and Chl, chlorogenic acid. Dotted discontinuous line marks 120% in OD_560 nm_ (%).

The MTT results indicated a large dose-dependent increase in mitochondrial respiration rate for gallic acid, which was not mirrored in cell count. To determine whether gallic acid and chlorogenic acid were possibly interfering in the MTT reaction due to their reducing properties, the MTT reaction was conducted by emulating a regular MTT assay (concentration and volumes of reagents), with some key differences: in all cases, PBS 1X was used (filter-sterilized and pre-heated at 37 °C), and no fermentation media or fecal slurries were added. Results indicate that both gallic acid and chlorogenic acid increase the absorbance at 560 nm compared to the vehicle (PBS 1X) above 120% in a dose-dependent way, demonstrating that these compounds reduce 3-(4,5-dimethylthiazol-2-yl)-2,5-diphenyl-tetrazolium bromide to insoluble formazan (Figure 12), mimicking the activity of viable mitochondria in this assay. However, only gallic acid 1–10 mM was statistically different (*p* < 0.0001) from PBS 1X (one-way ANOVA with Dunnett’s post hoc test). Choline 100 µM and DMB 10 mM (optimized conditions) did not alter formazan production when compared to the vehicle.

## 4. Discussion

Elevated TMAO circulating levels have been associated with CVD and atherosclerosis development [4,5,6], and their reduction has been shown to produce cardioprotective and anti-atherosclerotic effects [11]. The inhibition of TMAO formation can be achieved by reducing the conversion of choline and carnitine to TMA in the gut, thus reducing the amount of TMA available for oxidation to TMAO [11]. Phytochemicals are known to exert cardioprotective and anti-atherosclerotic functions [13,27], and some of these compounds have also been reported to reduce TMAO formation [11,14,15,16,17]. It is thus possible that phytochemical inhibition of TMA formation may be an effective cardioprotective mechanism via lowering TMAO. However, so far there have been relatively few studies in this area, the majority of which are in animal models, which are inefficient for rapidly screening large numbers of potentially effective compounds [10]. Thus, the development of a high-throughput in vitro screening method to evaluate the inhibition of TMA formation in the gut by phytochemicals could be highly useful to select promising lead compounds, for subsequent focused in vivo studies. In this study, we performed in vitro anaerobic fecal fermentations using fecal samples from healthy humans and optimized parameters including the proportion of fecal slurry, choline concentration, fermentation time, positive control (DMB) concentrations, and concentration range of relevant phytochemicals (gallic acid and chlorogenic acid).

To reach our goal, we first studied choline (0–100 µM) utilization and TMA production in fermentations containing 5–45% fecal slurry (1:10 in PBS 1X) for 36 h (Figure 1 and Figure 2). Choline consumption curves indicated that choline-metabolizing bacteria became active around 6 h after fecal slurry inoculation, and that bacterial choline use occurred predominantly from 6 to 12 h. This is also corroborated by observed TMA production, which started to increase at 6 h in fecal slurry concentrations ≤ 20%. The plateau in TMA concentrations reached by fecal slurry concentrations ≥ 10%, as well as the final similar TMA concentration reached by each level of initial choline conditions regardless of fecal slurry concentration, demonstrate that all compounds capable of being metabolized into TMA were generally consumed and oxidized within the 36-h timeframe. Background (i.e., when 0 µM choline was added) concentrations of TMA production reached significant levels in all cases (>200 µM), suggesting that there are significant TMA precursors in the background (media and/or fecal samples). Indeed, TMA precursors have been reported in fermentation growth media, human fecal starters [20] and human feces [28]. In our study, when fermentation media + fecal slurry 1:10 (20%) was screened for TMA precursors, it showed ~225 µM choline and ~1120 µM betaine, both of which could contribute to background TMA production. Furthermore, endogenous phospholipids likely contributed to background TMA production. Based on background TMA production levels, it seems that endogenous choline is the primary compound giving rise to TMA background levels. For example, background levels in fecal fermentations with 20% fecal slurry report ~225 µM choline (Table 1), and TMA production at 36 h is quite proportional (~250 µM TMA). Thus, background production of TMA can likely be attributed predominantly to background choline levels. Oddly, the administration of 100 µM exogenous choline did not result in a proportional increase in TMA levels when compared to the background. For example, at 36 h in fecal slurry 20%, TMA levels reached by choline-free and choline 100 µM were, respectively, of 247.7 ± 2.9 µM and 405.6 ± 12.5 µM (a difference of ~158 µM), when we would have expected a maximal possible increase of ~100 µM in TMA between choline 100 µM and choline-free conditions (as 1 mol choline produces a maximum of 1 mol TMA). This is, however, in line with the study of Bresciani et al. [20]. When an omnivore human fecal starter was used to ferment 100 µM choline, TMA reached concentrations > 500 µM (initial background concentrations of choline of 45.6 ± 17.7 µM). Presumably, the addition of choline might cause bacterial shifts to promote the use of choline and other TMA precursors, causing an increase in TMA production capacity, and the lack of proportionality between the background and choline-supplemented conditions (i.e., differences are not just due to increases in substrate, but also increases in metabolic capacity, enhancing TMA production from background substrates as well). Overall, the speed of the changes in choline and TMA concentrations, and the time at which TMA concentrations reached a plateau were dependent on fecal slurry concentrations: the higher the fecal slurry concentration, the earlier the TMA concentration plateau was reached and the faster the kinetics. This suggests that choline oxidation of TMA is mediated by bacteria rather than spontaneous chemical oxidation.

To study the inhibition of choline microbial conversion to TMA in an in vitro assay, TMA production should be significantly different from the background in the assay. Choline concentrations of 5 and 10 µM were discarded because of their lack of statistical difference in kinetic curve and AUCs compared to the background. Although choline concentrations of 25 and 50 µM generated statistically different kinetic curves and AUCs against the background, the absolute differences in TMA production in at 36 h, and at time to reach TMA plateau were low. For example, in fecal slurry 10%, at 20 h (time to reach plateau in TMA) and at 36 h, the fold-change in TMA production between choline 25 and 50 µM and the background ranged between 1.1 and 1.3. However, this fold-change ranged between 1.4 and 1.6 in choline 100 µM. Due to the fact that the 5% fecal slurry percentage did not report a TMA concentration plateau, and that fecal slurry 45% required a high proportion of fecal slurry and reported the fastest kinetics, both conditions were not selected for further experiments. Fast kinetics might involve practical issues (missing relevant sample collection timepoints), and slower kinetics are also inconvenient due to longer experimental conditions. For these reasons, fecal slurry 20% was preferred over 10%. Thus, we selected 20% fecal slurry (1:10 in PBS 1X) and choline 100 µM for further experiments. Additionally, based on the observed TMA plateau profile, fermentation time was shortened to 24 h in follow-up experiments. These experimental conditions are in line with the ones used in similar fermentation experiments [11,20].

The first set of experiments was used to select optimal choline and fecal slurry fermentation conditions. Some questions still remained. For example, does non-microbial conversion of choline contribute significantly to TMA formation in this model? To study this contribution, choline (100 µM) was incubated in fermentation media (45% growth media and 55% PBS 1X) for 24 h under anaerobic conditions in the absence of fecal slurry, which was substituted by PBS 1X (Figure 3). Overall, choline concentrations remained > 70% of initial concentration throughout the incubation, and spontaneous TMA production barely occurred (reaching 3.07 ± 0.28 µM at time 24 h, a small fraction of TMA production observed during fecal incubations). Thus, the vast majority of observed TMA formation in fermentations with fecal slurry does in fact appear to originate from the action of the microbiota on relevant substrates. Nevertheless, the fecal fermentation inoculated with fecal slurry itself contains choline and betaine, and may even contain other TMA precursors such as phosphatidyl choline, which can be metabolized to TMA [7,8,26]. To corroborate that TMA was in fact produced from the exogenous choline rather than only endogenous background choline levels, choline and TMA were tracked for 24 h in fermentations containing 20% fecal slurry (1:10 in PBS 1X) with and without choline added at 100 µM (Figure 4). TMA production from the background reached ~245 µM, which further demonstrates that the presence of TMA-producing substrates in background fermentation conditions, understood as everything except added choline (growth media + PBS + fecal slurries), contributes significantly to TMA production. This is in line with our previous results and the study of Bresciani et al. [20]. Of note, we reported relevant concentrations of choline and betaine in our background, suggesting that fecal slurries and media contribute to TMA formation through intrinsic metabolism of endogenous choline, and potentially betaine. Other compounds, such as L-carnitine [8,26] or TMAO through retro-conversion to TMA [25], seem not to contribute to TMA formation in the background as they are not detected in fermentation media + fecal slurry. The differences in TMA production between the choline-free and the choline-supplemented conditions (~154 µM) indicate that exogenous choline is being oxidized into TMA by the action of the bacteria present in the fecal slurry. The lack of proportionality (154 µM increase in TMA from addition of 100 µM choline) again suggests that the addition of exogenous choline promotes a shift in bacteria communities towards a higher TMA-producing profile, which would explain the lack of proportionality between the levels of choline added and the generated TMA compared to the background. Thus, further studies will include the analysis of background conditions.

A range of DMB concentrations between 0.2 and 10 mM were tested to optimize the concentrations of a known TMA-lyase inhibitor (Figure 5). This range was selected based on preliminary experiments (data not shown). The kinetic curves corroborate our previous results that the metabolism of choline by fecal slurry microbiota occurs primarily from 6 to 10 h in DMB-free conditions, and show that this occurs from 6 to 12 in DMB-supplemented conditions. Our data thus suggest that DMB is delaying the microbial oxidation of exogenous choline into TMA by inhibiting TMA-lyase enzymes. Previous studies using DMB have already reported such inhibitory effects. For example, DMB (0–2 mM) reduced TMA-lyase activity by ~20% in *E. coli* cells transfected with *D. alaskensis* CutC/D genes [11]. Of note, the microbial oxidation of exogenous choline occurred at up to 12 h (Figure 4A). Thus, the exogenous substrate is not present after this time, and any TMA formation after 12 h (Figure 4B) is originating from other TMA precursors. Considering all data, the DMB concentrations that demonstrate optimal performance as a TMA-lyase inhibitor are 2 and 10 mM.

The odd behavior of DMB 5 mM compared to 2 and 10 mM could be attributed to the higher initial measured choline concentration in that condition (Figure 5A), or to U- and/or J-shape dose–response curves (hormetic effect) [29]. DMB 5 mM seems to follow the trend of lower (1 and 0.2 mM) DMB concentrations. To consider this variability in the inhibitory effect of DMB, choline concentrations relativized to initial (0 h) choline concentrations were evaluated at relevant metabolically active time points (6–12 h) (Figure 5B). The higher the DMB concentration, the higher the percentage of choline detected, which indicates that choline is not being metabolized by bacteria, potentially due to a TMA-lyase inhibitory effect of DMB. To further investigate the inhibitory effect of DMB, we analyzed the relative change in TMA of DMB-treated groups in comparison to DMB-free conditions. At 12 h, a time point at which choline is no longer quantifiable (above the background) in any groups, DMB-treated groups report a >30% reduction in TMA concentration when compared to DMB-free conditions (Figure 5C), which is in line with the percentages of inhibition of 40–50% reported by Wang et al. [11] in human fecal intact cells. Overall, our results reinforce the idea that DMB inhibits choline metabolism, probably due to a TMA-lyase inhibitory effect already reported by Wang et al. [11].

It is clear now that DMB does inhibit TMA production, and this is linked with a reduced usage of choline. However, whether or not these changes in TMA and choline are attributed to a TMA-lyase inhibitory effect of DMB or a cytotoxic/cytostatic effect in our study are unknown. Wang et al. [11] have already defined DMB as a TMA-lyase inhibitor using cell lysates, intact cells, and microbiota from mice and humans. In their study, they also corroborated that the growth of certain bacterial strains (*P. mirabillis*, *P. penneri* and *E. fergusonii*) under ~8 mM DMB was not compromised. We analyzed the cell content and mitochondrial respiration rate of our fermentations with or without DMB (0.2–10 mM) at the end (24 H) of the fermentations (Figure 6). No significant differences were reported between background and choline +/DMB-free conditions, which suggests that choline administration is not toxic to bacteria. Although DMB seems to have some slight effects on bacterial growth, it does not have a significant impact on biochemical activity. Overall, these results indicate that DMB administration to choline-supplemented bacteria does not alter their growth or biochemical activity in a way that could explain the observed changes in TMA and choline metabolism. Thus, DMB effect on TMA and choline concentrations does not predominantly arise from an inhibition of bacterial growth or toxic effect, which agrees with the TMA-lyase inhibitory effect reported by Wang et al. [11]. To our knowledge, this is the first attempt to study mitochondrial respiration rate in the context of choline microbial oxidation into TMA. However, these data represent the viability of the whole microbial species present in the fermentations. Overall, the highest inhibition of microbial choline oxidation into TMA with low effect on cell growth and mitochondrial respiration is the dose of 10 mM DMB. The inhibition of microbial choline oxidation into TMA through the DMB-mediated inhibition of TMA-lyases in human fecal samples is ~50% in our study (DMB 10 mM), and ~70% in the study of Wang et al. (DMB 2 mM). DMB can thus be used as a positive control in the inhibition of TMA-lyases. Thus, 10 mM DMB was selected as a positive TMA-lyase control treatment in further studies. However, its reported low levels in foodstuffs (maximum levels of 25 mM in balsamic vinegars, red wine and cold-pressed extra virgin olive oils [11]) make the inhibition of TMA-lyases virtually impossible in a normal diet.

Some phenolic compounds have shown their ability to reduce TMAO levels in different models [10]. Thus, we selected two phenolic compounds highly consumed by humans: gallic acid and chlorogenic acid [30]. Moreover, gallic acid as well as chlorogenic acid and their derivatives have reported relevant cardioprotective functions [31,32]. We thus studied the potential use of these two phenolic compounds to inhibit TMA production in a range of concentration within 0.1–10 mM at relevant time points (8 and 12 h). Both gallic acid and chlorogenic acid reported a dose-dependent effect on choline use and TMA production. The most effective doses were gallic acid 10 mM and chlorogenic acid 10 mM, both at reducing choline use and TMA production. Of note, the percentage of TMA production inhibition at 12 h was >80% for those conditions, which is superior to the results reported by DMB. Interestingly, TMA production at the 10 mM of gallic acid was equivalent to the background, and it was lower than the background for chlorogenic acid at 10 mM, indicating that chlorogenic acid inhibited background TMA production as well. Overall, between both compounds, chlorogenic acid seems to be a better inhibitor of TMA production. This is reflected in lower IC_50_ for chlorogenic at 8 h. Remarkably, these inhibitory effects were not attributed to a reduction in cell number, as cell count data demonstrate a lack of significant reduction in growth between phenolic compound-treated fermentations compared to choline-free and choline 100 µM conditions. Due to gallic acid and chlorogenic acid interference with MTT, cell viability in these specific conditions could not be accurately assessed. The greater MTT-reactive activity of gallic acid compared to chlorogenic acid reflects the superior reducing activity of gallic acid, due to its three phenolic hydroxyl groups on the same ring. Due to the antioxidant/reducing activities of many phytochemicals (particularly phenolics), MTT will not be a generally valid approach to assess cellular viability when these compounds are present. Other approaches of assessing cell viability will need to be employed.

To further study the effect of gallic acid and chlorogenic acid on TMA production inhibition, other potential sources of TMA were studied. Besides choline, L-carnitine, γ-butyrobetaine and TMAO were not detected or not quantified at 0 h in all samples. However, betaine was present in fermentation media. Betaine levels were monitored in this third experiment, and showed interesting results. Given the high concentration of betaine in fermentation media, its quantification through the addition of standard betaine would provide inaccurate values. Rather, we monitored the relative changes of betaine in fermentation conditions relativized to choline 100 µM fermentation conditions. We also plotted relativized betaine/betaine-d_9_ peak areas. Of note, no differences were reported at time 0 h between treatment groups, but relative betaine levels versus choline-free (0 µM) increased over time in gallic acid- and chlorogenic acid-supplemented groups. This effect was most relevant for chlorogenic acid treatments, especially at 8 h (all concentrations) and at 12 h for 5 mM. Choline is primarily metabolized to TMA through its direct oxidation mediated by the bacterial CutC/D enzyme complex [7]. Alternatively, choline can be metabolized to TMA in a series of catalytic steps involving choline dehydrogenase, betaine aldehyde dehydrogenase and betaine reductase, which generate betaine as an intermediate product [33]. Our data seem to indicate that gallic acid, and especially chlorogenic acid, could be modulating the metabolic route by which choline is metabolized, which could potentially indicate a TMA-lyase inhibitory effect of these compounds. DMB, however, is not reporting these effects. Substrate analogues tend to inhibit enzymes by competing for enzyme’s active site. This is the case of a cyclic choline analogue that inhibits CutC [34], and in silico docking studies have suggested this as a potential mechanism of action of some other synthetic choline analogues [18]. However, other compounds can inhibit enzymes by interacting with other enzyme pockets and shifting enzyme structure in a way that it loses enzymatic activity. Thus, this could be a potential explanation between the differences in betaine production between DMB and the tested phenolic compounds when compared to choline 100 µM, as DMB is structurally very close to both choline and betaine. The shunting of choline to betaine by phenolic compounds warrants further investigation.

Overall, gallic acid and chlorogenic acid reduce TMA formation, either through a direct TMA-lyase inhibitory effect or another non-toxic one. In fact, the data presented herein do not prove that gallic acid and/or chlorogenic acid reduce TMA production through direct TMA-lyase inhibitory effects. For example, in the study of Bresciani et al. [20], the most potent treatments to reduce TMA production were those that involved the supplementation of growth media with mono or disaccharides (i.e., sucrose, fructose and glucose). This suggests that some treatments reduce TMA production simply by providing better energy substrates than choline. Put simply, in our experiment, bacteria could be using gallic acid and chlorogenic acid as a more preferent source of energy than choline, thus leading to a reduction in choline use and TMA production. Indeed, both gallic acid and chlorogenic acid can be metabolized by the gut microbiota [35,36]. For example, chlorogenic acid can be rapidly metabolized (7 h) in human fecal fermentation into 3-(3,4-dihydroxyphenyl)propionic acid [35], which opens the door to new mechanistic questions such as what is the bioactive compound, chlorogenic acid or one of its microbial metabolites. Of note, the fact that IC_50_ for chlorogenic acid is higher at 12 h than at 8 h could indicate that chlorogenic acid is being degraded to a more potent inhibitory metabolite. Moreover, these compounds could also inhibit choline bacterial uptake. Future studies using cell lysates and the analysis of choline and TMA within bacterial cell should shed some light onto those mechanisms. Furthermore, the mechanisms of action may be to reduce the abundance of bacterial species that express the TMA-lyase gene. Further studies are necessary in order to investigate this possibility.

Concentrations of gallic acid and chlorogenic acid at 5–10 mM have herein been demonstrated to be efficient at inhibiting the microbial conversion of choline into TMA in vitro, although the mechanisms by which this occurs are still unknown. Although these doses can be perceived as high for phenolic compounds, in the context of TMA production inhibition and selected phenolic compounds, those doses are likely relevant. First, the poor bioavailability and extensive phase-II metabolism of phenolics, which is a main limiting factor in systemic bioavailability and effects in peripheral organs and tissues such as the liver and adipose tissue of these compounds [37], are not anticipated to play a crucial limiting role in the inhibition of choline microbial conversion into TMA. Indeed, over 95% of ingested phenolic compounds reach the colon lumen [38], which is the localization where their target of this particular bioactivity is located: the gut microbiota. Thus, considering the systemic low bioavailability of phenolic compounds, gallic acid and chlorogenic reach this specific target destination at the highest possible doses achieved anywhere in the body. Second, gallic acid and chlorogenic acid, as well as their derivates, can be found in high concentrations in commonly consumed fruits and vegetables [39,40], and the consumption of these compounds is high [30]. For example, coffee is a rich source of chlorogenic acid (a cup of coffee of ~200 mL contains between 50 and 150 mg chlorogenic acid) [41], and ~65% of all phenolic compounds consumed by coffee consumers come from coffee [42]. Although large differences exist in human colon volumes, the mean value approximates to 560 mL [43]. This suggests that average chlorogenic acid concentrations, at least in the proximal colon where chlorogenic acid is starting to be metabolized by the gut microbiota and right after its arrival, could range up to 0.25–0.76 mM after consumption of a single cup of coffee. Daily gallic acid consumption has been estimated at 25 mg/day [44], which suggests that the maximum concentration could be 0.26 mM. However, gallic acid can be produced by the gut microbiota metabolism of gallated flavan-3-ols and anthocyanins [45,46,47]. Tea contains both gallic acid and gallated flavan-3-ols, as well as lower quantities of chlorogenic acid [48]. For coffee and tea consumers, ~80% of all phenolic compounds consumed come from these two beverages [49]. It thus seems that concentrations on the lower millimolar scale, such as those determined to inhibit microbial TMA production from choline, are on the order of physiologically relevant concentrations achieved in the colon. Furthermore, common food-derived supplements (functional foods supplemented with chlorogenic acid and/or gallic acid) as well as nutraceuticals could easily achieve the levels employed here.

Overall, we have reported that: (1) DMB 10 mM can be used as a control substance in TMA production inhibition studies; (2) gallic acid and chlorogenic acid (10 mM) are phenolic compounds able to reduce TMA formation at a higher proportion than DMB 10 mM; and (3) the effects reported by DMB, gallic acid and chlorogenic acid (10 mM) are due to non-toxic effects within a period of 12 h. These results open the door to using this developed methodology to study other phenolic compounds in a high-throughput way. However, the main limitation of this study is the fecal slurry itself. Further studies using fecal starters from high TMAO and/or TMA producers should be selected to study the inhibition potential of phenolic compounds, or any other treatments for that matter. Additionally, the source of the fecal slurry can have a great impact on other parameters, such as capacity to metabolize phenolic compounds such as chlorogenic acid [35,50], which ultimately could affect choline microbial conversion to TMA. The concentrations of betaine and choline in the fermentation media + fecal slurry are also a drawback in this experimental design. The quantification of choline in all samples in this study was achieved by spiking fermentation media (45% growth media +55% PBS 1X) without fecal slurry with different concentrations of choline to prepare external standard curves. Given the presence of choline in the fermentation media (~225 µM), only exogenously added choline was quantifiable. Thus, the results presented herein do not take into account endogenous choline levels. For example, when we report 100 µM choline in fermentations containing 20% fecal slurry, the background levels have been subtracted, and the real concentration would approximate to 325 µM total choline. This effect was not reported for TMA, as TMA levels were non-detectable in the original fecal slurry and the fermentation media inoculated with fecal slurry 1:10 (20%). This was also true for L-carnitine, γ-butyrobetaine and TMAO, but not for betaine. As a matter of fact, betaine levels were so high in the fecal fermentation inoculated with the fecal slurry 1:10 (20%) that addition of betaine would not produce a linear curve, hence making its absolute quantification problematic. Simply running calibration curves in water does not take into account the matrix effect, which could modulate the quantification of choline and betaine. Rather, calibration curves should be constructed with a matrix as similar as possible. To potentially bypass this issue, results could be presented as percentage of change versus background analyte concentrations, as performed by Bresciani et al. [20]; use deuterated choline, as performed by Wang et al. [11]; or study the inhibitory effect of phytochemicals on background choline microbial metabolism into TMA. Finally, other cell viability tests than MTT should be considered for screening studies.

The method presented herein facilitates efficient, rapid screening of large compounds libraries for potential TMA-reducing activity. This will allow rational selection of particularly effective lead compounds for focused animal studies, potentially leading to the development of strategies to benefit specific population groups with elevated TMAO circulating levels. This approach should prove much more effective at identifying lead compounds for human trials, as opposed to the scattered approach that is currently being employed.

## 5. Conclusions

In this study, fecal slurry (20%), choline concentrations (100 µM), reference TMA-lyase inhibitor concentrations (DMB 10 mM), and fermentation time (12 h) have been optimized to study the inhibition of TMA production by phenolic compounds. Gallic acid and chlorogenic acid were identified as TMA production inhibitors at concentrations of ~5 mM, which demonstrates that this methodology can be applied to screen for lead phenolic compounds with TMA production inhibition properties. Given the 96-well plate format (fermentation volume of 0.9 mL), this can be used to screen for compounds in a high-throughput manner.

## Figures and Tables

**Table 1:** Concentration of TMA and TMA-related compounds in fecal slurry and fermentation background (fermentation media + fecal slurry 1:10 (20%)).

Compound	Fecal Slurry (µM)	Fermentation Background (µM) ^a^
Choline	81.96 ± 3.54	226.77 ± 2.42
L-carnitine	n.d.	n.d.
Betaine	248.05 ± 5.08	1118.54 ± 3.95
γ-Butyrobetaine	n.d.	n.d.
TMAO	n.d.	n.d.
TMA	n.d.	n.d.

Results are expressed as mean (µM) ± SEM (*n* = 4). Abbreviations: TMAO, trimethylamine N-oxide; TMA, trimethylamine; and n.d., not detected. ^a^ Fermentation background was composed of 45% growth media and 55% PBS 1X, including a final concentration of fecal slurry (1:10 in PBS 1X) of 20%.

## Data Availability

The authors will make data available upon request.

## Supplementary Materials

The following are available online at https://www.mdpi.com/article/10.3390/nu13051466/s1, Figure S1: Effect of gallic acid and chlorogenic acid (A,B) in the peak area of betaine relativized to its internal standard betaine-d_9_. Relative changes in betaine/betaine-d_9_ versus choline 100 µM conditions (C, D). All fermentations were carried out at optimal conditions (fecal slurry 20% and choline 100 µM). Results are expressed as% ± SEM (*n* = 6). Different letters indicate statistical differences (*p* < 0.05) by One-way (C,D) or Two-way (A,B) ANOVA (Tukey’s post hoc test). Factors for Two-way ANOVA were gallic acid or chlorogenic acid concentration and time. Abbreviations: DMB, 3,3-dimethyl-1-butanol; GA, gallic acid; and Chl, chlorogenic acid. Table S1: Molecular weight (MW), retention time (RT) and optimized MRM condition for TMA and related compounds; Table S2: Parameters for the quantification of TMA and TMA-related compounds in spiked fecal fermentation samples by UPLC-MS/MS.
