# Supplementary material for: Development of a High-Throughput Method to Study the Inhibitory Effect of Phytochemicals on Trimethylamine Formation
Source: Nutrients. 2021 Apr 26;13(5):1466. doi: 10.3390/nu13051466 (PMC8145906; doi:10.3390/nu13051466)
Supplement: Supplementary file 1 [file nutrients-13-01466-s001.zip › nutrients-1190241-supplementary.pdf]

Supplementary Materials

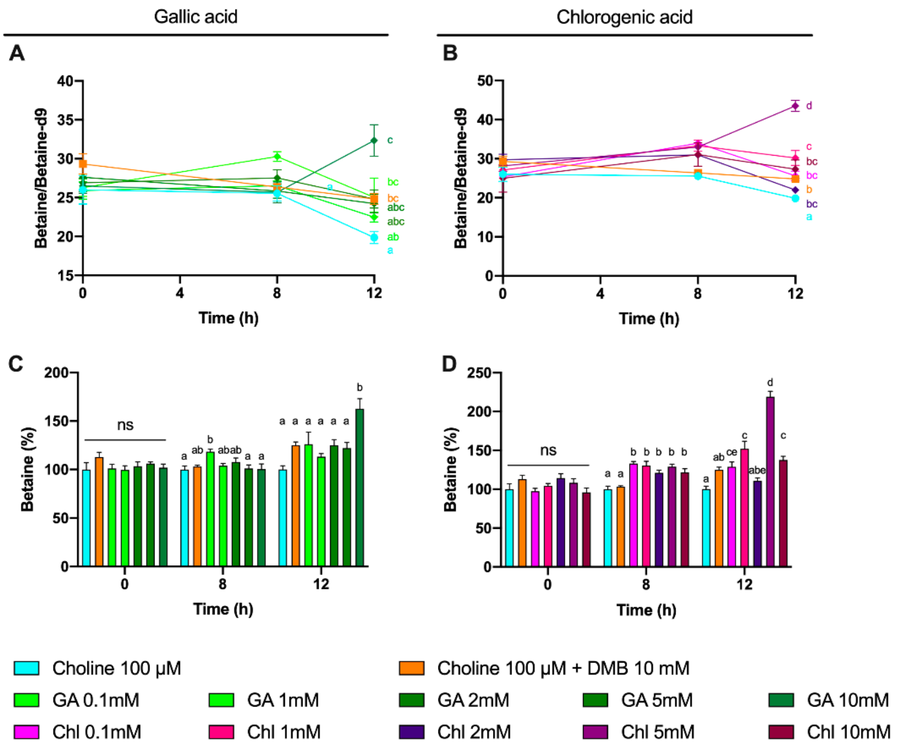

Figure S1: Effect of gallic acid and chlorogenic acid (**A**,**B**) in the peak area of betaine relativized to its internal standard betaine-d_9. Relative changes in betaine/betaine-d_9 versus choline 100 µM conditions (**C**,**D**). All fermentations were carried out at optimal conditions (fecal slurry 20 % and choline 100 µM). Results are expressed as % ± SEM (*n*=6). Different letters indicate statistical differences ($p<0.05$) by One-way (**C**,**D**) or Two-way (**A**,**B**) ANOVA (Tukey's post hoc test). Factors for Two-way ANOVA were gallic acid or chlorogenic acid concentration and time. Abbreviations: DMB, 3,3-dimethyl-1-butanol; GA, gallic acid; and Chl, chlorogenic acid.

Table S1. Molecular weight (MW), retention time (RT) and optimized MRM condition for TMA and related compounds.

Compound	MW	RT (min)	MS/MS Transition	CV (V)	CE (eV)
Choline	103.16	1.01	104.2>60.0	38	16
Choline-d_9	112.16	1.02	113.3>69.1	40	16
L-carnitine	161.20	2.17	162.3>85.0	34	20
L-carnitine-d_9	170.25	2.17	171.3>85.0	34	20
Betaine	117.15	1.07	118.2>59.4	44	18
Betaine-d_9	126.15	1.07	127.3>68.1	46	18
γ-Butyrobetaine	145.20	2.26	146.3>87.0	26	16
TMAO	75.11	1.35	76.2>58.9	40	10
TMAO-d_9	84.12	1.39	85.2>68.1	40	12
TMA [a]	59.11	0.76	146.3>118.2	34	16
TMA-d_9 [a]	68.17	0.75	155.3>127.2	34	20

Abbreviations: TMAO, trimethylamine *N*-oxide; TMA, trimethylamine; MW, molecular weight; RT, retention time CV, cone voltage; and CE, collision energy. [a] Molecular weight listed is for the original molecules, and MS/MS transitions listed for derivatives (ethyl betaine and ethyl betaine-d_9).

Table S2: Parameters for the quantification of TMA and TMA-related compounds in spiked fecal fermentation samples by UPLC-MS/MS.

Compound	Calibration curve	R^2	Working linearity range (µM)	LOD (µM)	LOQ (µM)	MDL (µM) [a]	MQL (µM) [a]
Choline	y = 0.765x + 3.003	0.997	1.93 – 247.10	0.488	1.628	0.977	3.256
L-carnitine	y = 5.932x – 4.723	0.997	0.24 – 497.72	0.024	0.081	0.049	0.162
γ-Butyrobetaine	y = 0.197x + 0.181	0.998	0.27 – 37.92	0.032	0.105	0.063	0.210
TMAO	y = 0.355x – 0.978	0.997	0.43 – 228.71	0.011	0.037	0.075	0.075
TMA	y = 58.301x + 13.776	0.999	0.84 – 462.73	0.194	0.648	0.387	1.291

Abbreviations: TMAO, trimethylamine N-oxide; TMA, trimethylamine; R^2, determination coefficient; LOD, limit of detection; LOQ, limit of quantification; MDL, method detection limit; and MQL, method quantification limit. [a] MDL and MQL for 25 µL of fecal fermentation media.
